# Nestin expression in the cell lines derived from glioblastoma multiforme

**DOI:** 10.1186/1471-2407-6-32

**Published:** 2006-02-02

**Authors:** Renata Veselska, Petr Kuglik, Pavel Cejpek, Hana Svachova, Jakub Neradil, Tomas Loja, Jirina Relichova

**Affiliations:** 1 Cell Culture Laboratory, Department of Biology, School of Medicine, Masaryk University in Brno, Czech Republic; 2 Laboratory of Molecular Cytogenetics, Department of Genetics and Molecular Biology, School of Science, Masaryk University in Brno, Czech Republic; 3 Department of Neurosurgery, School of Medicine, Masaryk University in Brno, Czech Republic

## Abstract

**Background:**

Nestin is a protein belonging to class VI of intermediate filaments that is produced in stem/progenitor cells in the mammalian CNS during development and is consecutively replaced by other intermediate filament proteins (neurofilaments, GFAP). Down-regulated nestin may be re-expressed in the adult organism under certain pathological conditions (brain injury, ischemia, inflammation, neoplastic transformation). Our work focused on a detailed study of the nestin cytoskeleton in cell lines derived from glioblastoma multiforme, because re-expression of nestin together with down-regulation of GFAP has been previously reported in this type of brain tumor.

**Methods:**

Two cell lines were derived from the tumor tissue of patients treated for glioblastoma multiforme. Nestin and other cytoskeletal proteins were visualized using imunocytochemical methods: indirect immunofluorescence and immunogold-labelling.

**Results:**

Using epifluorescence and confocal microscopy, we described the morphology of nestin-positive intermediate filaments in glioblastoma cells of both primary cultures and the derived cell lines, as well as the reorganization of nestin during mitosis. Our most important result came through transmission electron microscopy and provided clear evidence that nestin is present in the cell nucleus.

**Conclusion:**

Detailed information concerning the pattern of the nestin cytoskeleton in glioblastoma cell lines and especially the demonstration of nestin in the nucleus represent an important background for further studies of nestin re-expression in relationship to tumor malignancy and invasive potential.

## Background

Nestin was originally described as an antigen of monoclonal antibody RAT401 against embryonic spinal cord [1] and was subsequently identified as a class VI intermediate filament protein [2], which is closely related to the neurofilament branch [3]. Nestin expression has been proved in both rodent and human neural stem cells in various areas of the developing CNS as well as in immortalized stem cell and precursor cell lines [4-7]. During the development of the mammalian CNS, expression of cytoplasmic intermediate filaments begins with cytokeratins in early embryonic cells, through nestin and vimentin in proliferating neuroepithelium to the neurofilaments and GFAP in differentiated neurons and astrocytes, respectively [3].

In the adult CNS, nestin is expressed only in stem cells of the subventricular zone and to a lesser extent in the choroid plexus [6]. In the normal adult human brain, several morphological types of nestin-positive cells (neuron-like, astrocyte-like, cells with smaller cell bodies and fewer processes) are detectable in different areas of forebrain [8]. Re-expression of down-regulated nestin was demonstrated in reactive astrocytes following certain types of brain injuries. This reversion to the immature phenotype may serve to protect the cells, perhaps by making them less susceptible to the attendant hypoxia that can occur after injury [9]. Similarly, re-induction of nestin has been reported in reactive astrocytes and endothelial cells in cerebral abscesses, this process is probably caused by pathogenic microorganisms inducing inflammatory stress in the tissue [10].

Cell-specific expression of intermediate filament proteins in normal tissue and the differences in this expression in tumors represent an important tool for tumor diagnostics. From this point of view, immunohistochemical and/or immunocytochemical examination of nestin may serve as a useful tool for classification and accurate grading of human malignancies; especially since this protein has been found in many kinds of tumors, predominantly in tumors originating from immature, stem or progenitor cells. Using immunohistochemical staining of paraffin-embedded tissue sections, nestin expression has been detected in brain tumors and tumors derived from CNS tissues, such as, neurocytomas, gangliogliomas, ependymomas, pilocytic astrocytomas, malignant gliomas including glioblastoma multiforme, primitive neuroectodermal tumors (PNETs), medulloblastomas and medulloepitheliomas [4,11-16]. Up-regulation of nestin has also been shown in rhabdomyosarcomas [17], gastrointestinal stromal tumors and interstitial cells of Cajal [18,19], as well as in metastatic melanomas [20].

For detailed studies of intermediate filament protein expression and regulation, several cell lines derived from astrocytic tumors were used [21,22]. The high-grade astrocytomas, i.e. anaplastic astrocytoma (WHO grade III) and glioblastoma multiforme (WHO grade IV), seem to be exceptionally suitable models for the investigation of nestin re-expression and its relationship to the other intermediate filament proteins. Significant changes in these proteins (particularly GFAP and nestin), which are associated with motility and invasiveness, have been described in the astrocytoma cell lines [21]. Other findings suggest that the regulation of GFAP and nestin expression occurs at the transcriptional level [22]. Even though there have been several studies that focused on the detection of nestin in both tumor and normal cells, there are few publications describing the morphology of nestin cytoskeleton in individual human tumor cells. Therefore, the detailed pattern of nestin-containing intermediate filaments as an integral part of cytoskeletal structures is still unclear.

The aim of this study was to investigate, at both the cellular and ultrastructural levels, the nestin cytoskeleton in individual cells of two cell lines derived from glioblastoma multiforme. We characterized the morphology of nestin-positive filaments during the cell cycle, as well as the intracellular distribution of nestin molecules in the cytoplasm and also in the cell nucleus.

## Methods

### Cell culture

To obtain cell cultures, biopsy samples were taken from patients surgically treated for glioblastoma multiforme. The samples were coded and processed in the laboratory in an anonymous manner. This project has been approved by the Ethics Committee of the University Hospital Brno, Czech Republic.

The specimens were briefly washed in 70% ethanol, followed by two washing in PBS; the specimens were then mechanically chopped into pieces about 2 mm in diameter. After disaggregation, the pieces of tissue were washed three more times in PBS, this time with centrifugation. Next they were seeded into 25 cm^2 ^cell culture flasks containing 1 ml of complete medium, i.e. in DMEM (PAA Laboratories, Linz, Austria) supplemented with 20% fetal calf serum (PAA), 2 mM glutamine and antibiotics: 100 IU/ml of penicillin and 100 μg/ml of streptomycin (BioWhittaker, Inc., Walkersville, MD, USA). The cells were cultivated under standard conditions at 37°C and in an atmosphere of 95% air : 5% CO_2_. Once the specimen pieces had attached, the volume of the medium was gradually increased to 5 ml over the next 48 hours. As soon as the outgrowing cells covered about 60% of the surface, they were trypsinized, diluted and transferred into a new flask. A similar procedure was used for further subcultivations of both cell lines, which were derived from the primary cultures.

The expression of intermediate filament proteins and the chromosomal abnormalities were analyzed at passages 3 through 5 for both cell lines; the detailed study of the nestin cytoskeleton morphology using epifluorescence microscopy was carried out between passages 6 and 9. Confocal microscopy and ultrastructural analysis were performed using the GM7 cell line at passages 15 through 22.

For the study of cytoskeletal structures, we used both epifluorescence and transmission electron microscopy. Cell suspensions with a concentration of 10^4 ^cells/ml were inoculated onto glass coverslips and grown under standard conditions for 24 hours before cytoskeleton staining.

### Visualization of cytoskeletal structures

The cells were rinsed in PBS and fixed using 3% para-formaldehyde (Sigma Chemical Co., St. Louis, MO, USA) in PBS for 20 minutes at room temperature. After washing in the same buffer, the cells were permeabilized with a 0.2% solution of Triton X-100 detergent (Sigma) in PBS for 1 minute at room temperature. They were then washed with PBS and incubated for 10 minutes with 2% BSA to block the nonspecific binding of secondary antibodies.

Both cytoskeletal structures, i.e. intermediate filaments and microtubules, were stained by indirect immunofluorescence. To label the intermediate filaments, as our primary antibodies we used mouse monoclonal anti-vimentin antibodies, clones VIM 13.2 and LN-6 (Sigma), mouse monoclonal anti-vimentin antibody (Exbio, Prague, Czech Republic), mouse monoclonal anti-GFAP antibody, clone G-A-5 (Sigma), rabbit polyclonal anti-GFAP antibody (Sigma), and mouse monoclonal human specific anti-nestin antibody (Chemicon International Inc., Temecula, CA, USA). Microtubules were labeled using mouse monoclonal anti-α-tubulin antibody TU-01 (Exbio). All primary antibodies were applied at 37°C for 1 hour. The cells were then rinsed three times in PBS and treated with the corresponding secondary antibodies: anti-mouse antibodies conjugated with FITC or TRITC (Sigma) and/or anti-rabbit antibody conjugated with TRITC (Sigma).

After the labeling procedure was completed, the cells were mounted onto glass slides using Vectashield mounting medium with DAPI (Vector Laboratories, Burlingame, CA, USA). The cells were then observed in a Leitz Laborlux epifluorescence microscope and fluorescence micrographs were recorded with either a Wild Leitz camera or a CCD SB-8 camera. The images were stored using Camera 1.1 software (Dept. of Biology, Masaryk University, Brno, Czech Republic). For the detailed studies of cytoskeleton morphology, we used an Olympus FluoView-500 confocal imaging system in combination with an inverted Olympus IX-81 microscope. Images were recorded using an Olympus DP70 CCD camera. The images were analyzed using analySIS FIVE software (Soft Imaging System GmbH, Muenster, Germany) and the Olympus FluoView Confocal Laser Scanning Microscope System 4.3.

### Transmission electron microscopy

For immunodetection of nestin in ultrathin sections, cells were rinsed in PBS and then fixed with 2% para-formaldehyde (Sigma) in PBS for 1 hour at room temperature. After washing in PBS and dehydration, the cells were embedded in LR White (Polysciences Inc., London, UK). Ultrathin sections were labeled on grids and nestin was detected using mouse monoclonal anti-nestin antibody (Chemicon) and anti-mouse gold particle conjugated secondary antibody (Sigma). After immunolabeling, the specimens were contrasted with 2.5% uranyl acetate (Lachema-Pliva, Brno, Czech Republic) for 20 minutes and with Reynolds' solution for 8 minutes at room temperature. The specimens were examined using a Morgagni 268(D) transmission electron microscope (FEI Company, Hillsboro, OR, USA). Images were recorded using a MegaView III CCD camera (Soft Imaging System) and analyzed using AnalySIS software (Soft Imaging System).

## Results

### Characterization of cell lines

The tumor character of the derived cell lines was verified by GTG-banding during short-term cultivation – between passages 2 and 4. The GM7 cell line was identified as being near-tetraploid with a large number of structural and numerical abnormalities. The GM10 cell line was described as being near-diploid. The number of chromosomes varied from 43 to 46 and chromosomes 3, 15, 19, 22 and Y were the ones most frequently found to be missing. Genetic changes in both cell lines were also confirmed using FISH and HR-CGH methods (data not shown).

The astrocytic origin of these cell lines was confirmed by indirect immunofluorescence: both vimentin- and GFAP-positive cells were observed. However, the pattern of the cytoskeleton was quite different. While vimentin intermediate filaments, displaying a typical morphology, were detected in a majority of the cells in the culture, only a small proportion of the cells exhibited GFAP-positive filamentous structures. Most of cells in the culture showed only a diffuse, and usually weak, fluorescent signal for GFAP.

**Figure 1:**
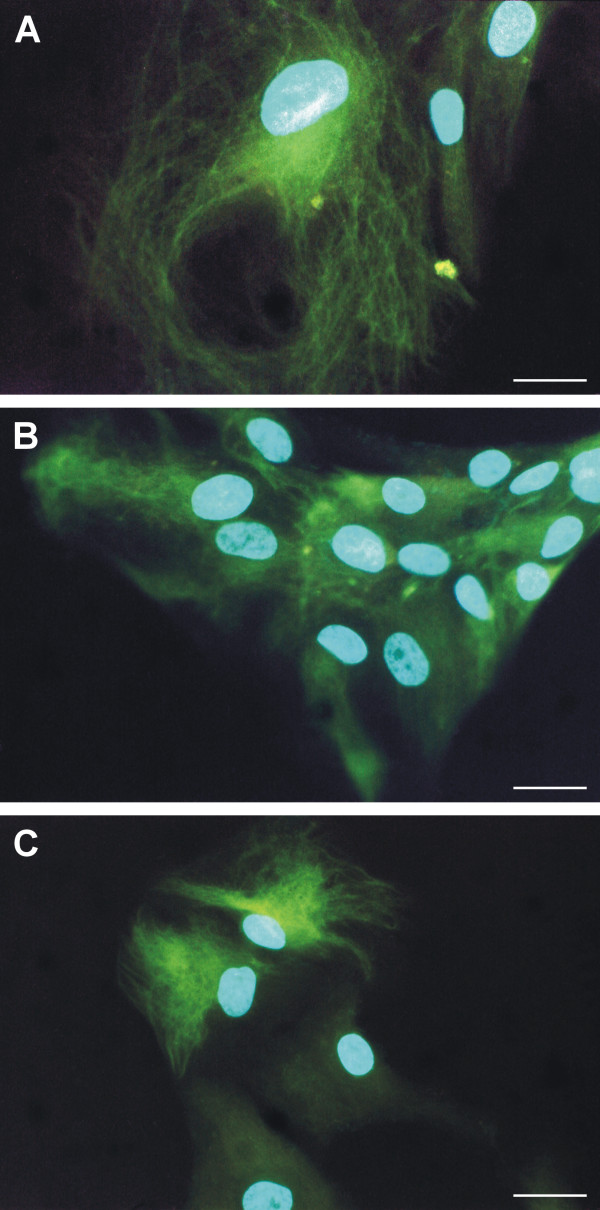
**Nestin expression in primary cultures of glioblastoma cells**. Nestin-positive intermediate filaments were detected in primary cultures in cells with different morphology – both in giant cells (A) and in smaller cells forming colonies (B). Due to heterogeneity of primary culture, non-tumor cells exhibiting no or very poor signals for nestin were also detectable together with nestin-positive cells (C). Nestin-positive filaments (A–C, green) stained by indirect immunofluorescence using FITC-labeled secondary antibody; nuclei labeled by DAPI (A–C, blue) are shown; bar, 25 μm.

In primary cultures, a different positivity for nestin was detected in the same cell population due to the mixture of various cell types (normal and transformed cells of astrocytic origin, endothelial cells) in the same culture. Transformed astrocytes were usually larger in size when compared to normal cells and, in addition to nestin positivity, abnormalities in nucleus morphology were also detected in these cells (Fig. 1A). Nestin expression and nestin-positive intermediate filament formation were also observed in the smaller cells forming monolayer (Fig. 1B). Nevertheless, nestin expression was very different in this cell population; it varied from no signal or a diffuse signal in the cytoplasm of, presumably, non-tumor cells up to characteristic nestin intermediate filaments as part of the transformed phenotype (Fig. 1C). During short-term cell cultures (between passages 2 and 4), a decreasing number of nestin-negative cells were observed. At passage 5, all cells in the population showed nestin positivity.

### Morphology of the nestin cytoskeleton

**Figure 2:**
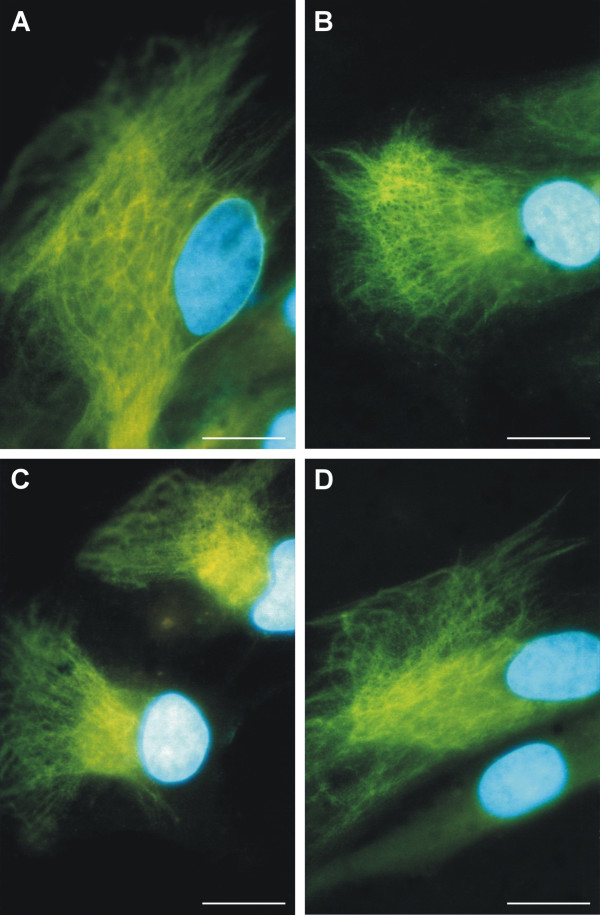
**Localization of nestin-positive intermediate filaments in glioblastoma cells**. A typical pattern of nestin-positive cytoskeleton in GM7 cells (A–D): the area with dense meshwork of nestin-positive filaments is located near the cell nucleus. Nestin-positive filaments (A–D, green) stained by indirect immunofluorescence using FITC-labeled secondary antibody; nuclei labeled by DAPI (A–D, blue) are shown; bar, 10 μm.

Cells in the monolayer expressing nestin-positive intermediate filaments usually showed their nuclei in asymmetric positions and the nestin formed a very dense meshwork of intermediate filaments either throughout the cytoplasm (Figure 2A) or only in a specific part of the cell (Figure 2B–D). In both situations, we were able to detect a region in the cytoplasm of each cell, which produced a strong signal for nestin and consisted of a dense meshwork of nestin-positive filaments. The area of this region was approximately the same as that of the nucleus and it was usually located in the vicinity of the nucleus; however, the region was not in the identical position as the cell nucleus (Figure 2A–D).

**Figure 3:**
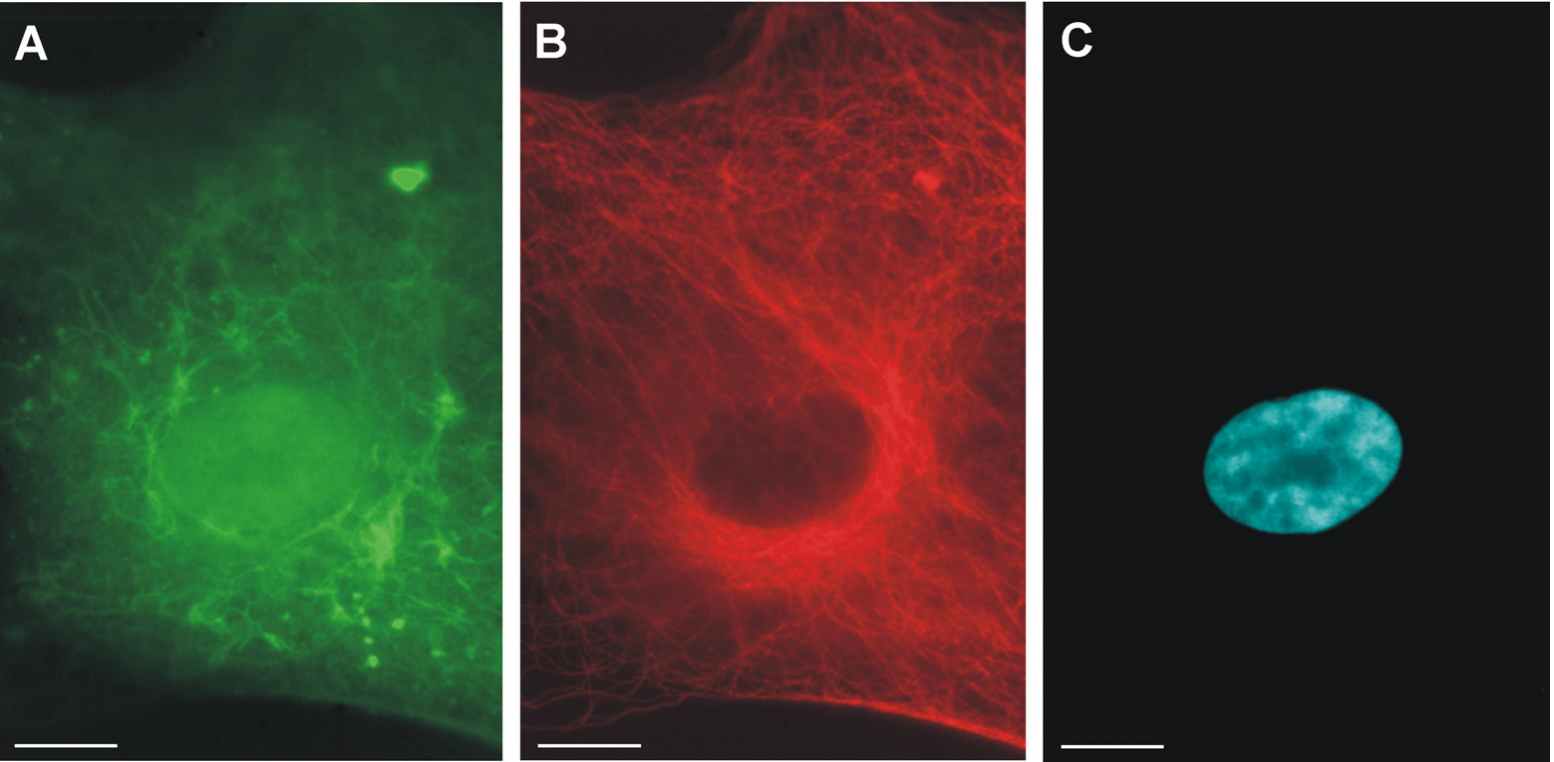
**Arrangement of nestin filaments and microtubules in the same cell**. Double labeling for both nestin (A) and tubulin (B) in the same cell of GM10 cell line showed no co-localization of nestin filaments with microtubules and confirmed different morphology of these cytoskeletal structures. Nestin-positive filaments (A, green) stained by indirect immunofluorescence using FITC-labeled secondary antibody; microtubules (B, red) stained by the same method using TRITC-labeled secondary antibody; nucleus labeled by DAPI (C, blue) are shown; bar, 10 μm.

To investigate a possible co-localization of this asymmetrical positioning of nestin filaments with a higher density of cytoplasmic microtubules in the MTOC region during interphase, a double labeling of both nestin and tubulin was performed. The results showed no co-localization of nestin filaments and microtubules and confirmed a different morphology of these cytoskeletal structures during interphase (Figure 3A–B).

**Figure 4:**
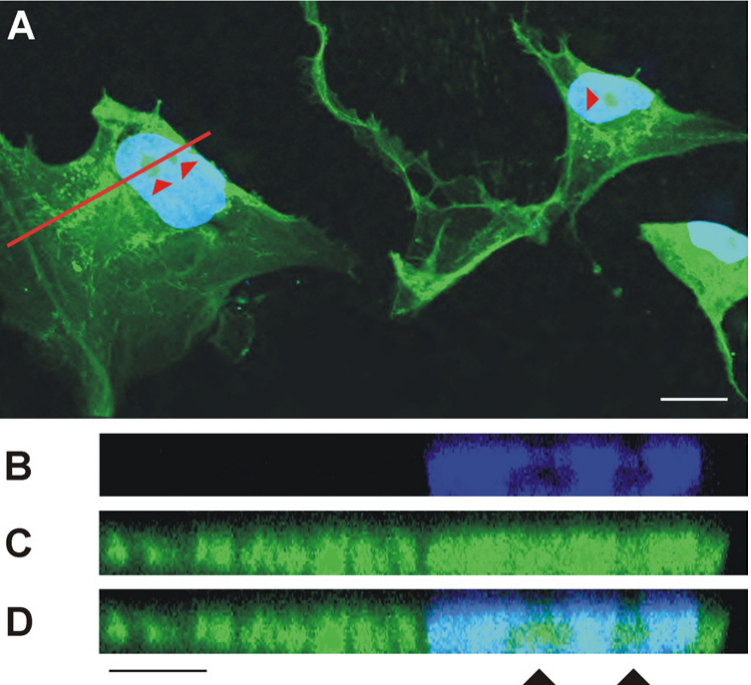
**Software cross-section through a cell with nestin-positive nucleus**. Nestin occurrence in cell nuclei was confirmed by confocal microscopy using software cross-section (B–D) through the selected cell (A, the plane of software cross-section is highlighted by the red line). The signal for nestin was detected also in the cell nucleus (C). The position of nucleoli is indicated by arrowheads. Nestin (A, C–D, green) stained by indirect immunofluorescence using FITC-labeled secondary antibody; nuclei labeled by DAPI (A–B, D, blue) are shown; bars, 20 μm (A), 10 μm (B–D).

In view of the nestin-positivity of the cell nuclei observed by fluorescence microscopy (Figure 3A), we performed software cross-sections through selected cells using confocal microscopy. The results showed that nestin was not only detectable in the cell nucleus, but also in the nucleoli (Figure 4A,C–D).

**Figure 5:**
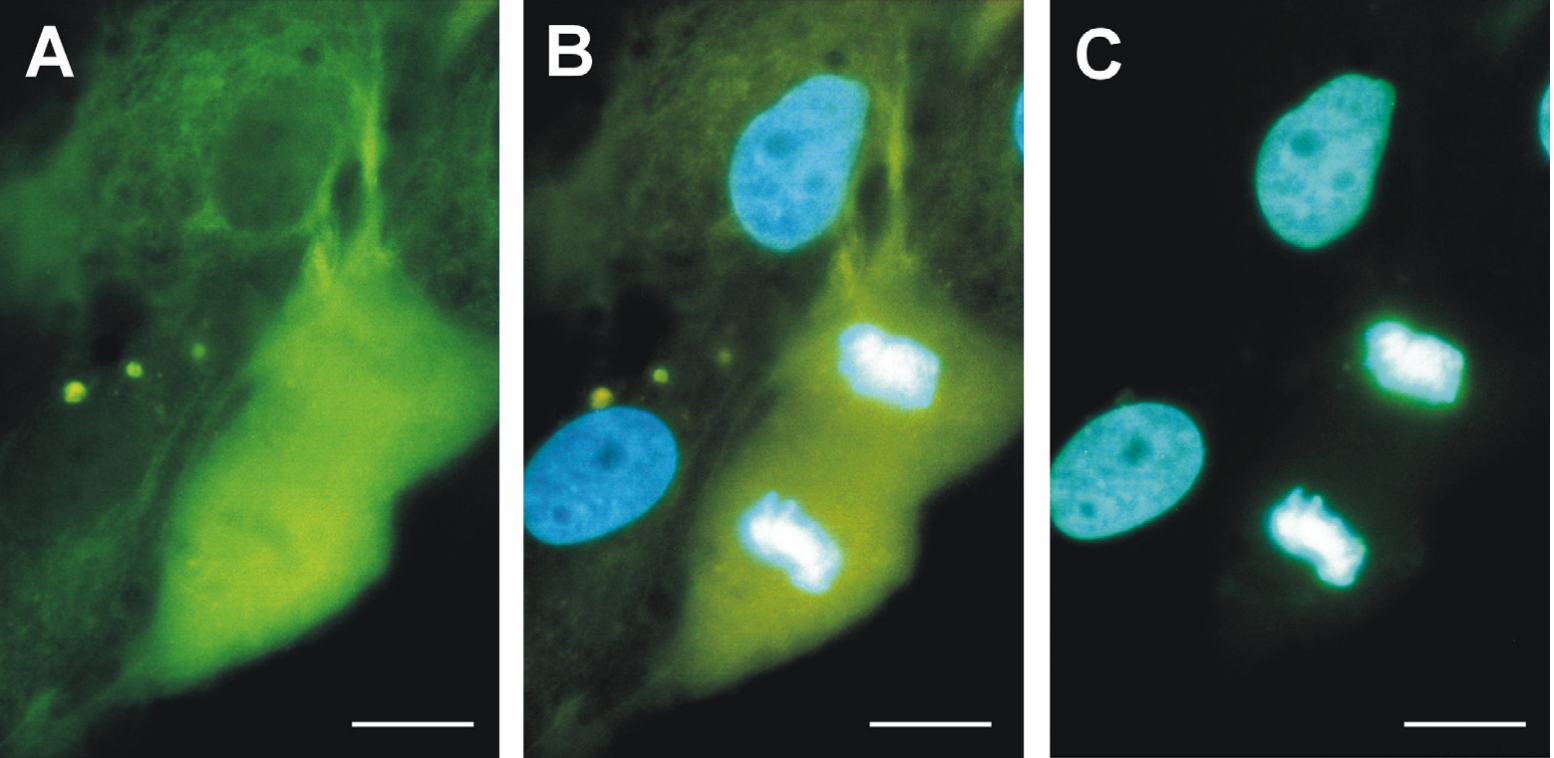
**Nestin in the mitotic cell**. Nestin-positive intermediate filaments were completely depolymerized and nestin was detectable as a strong diffuse fluorescent signal in the cytoplasm (A–B) of GM10 cell in late anaphase (B–C). Nestin (A–B, green) stained by indirect immunofluorescence using FITC-labeled secondary antibody; nuclei and chromosomes labeled by DAPI (B–C, blue) are shown; bar, 10 μm.

In mitotic cells, nestin intermediate filaments were completely depolymerized and as a result, nestin was detectable as a strong diffuse fluorescence in the cytoplasm of the cells (Figure 5A–C).

### Ultrastructural distribution of nestin

**Figure 6:**
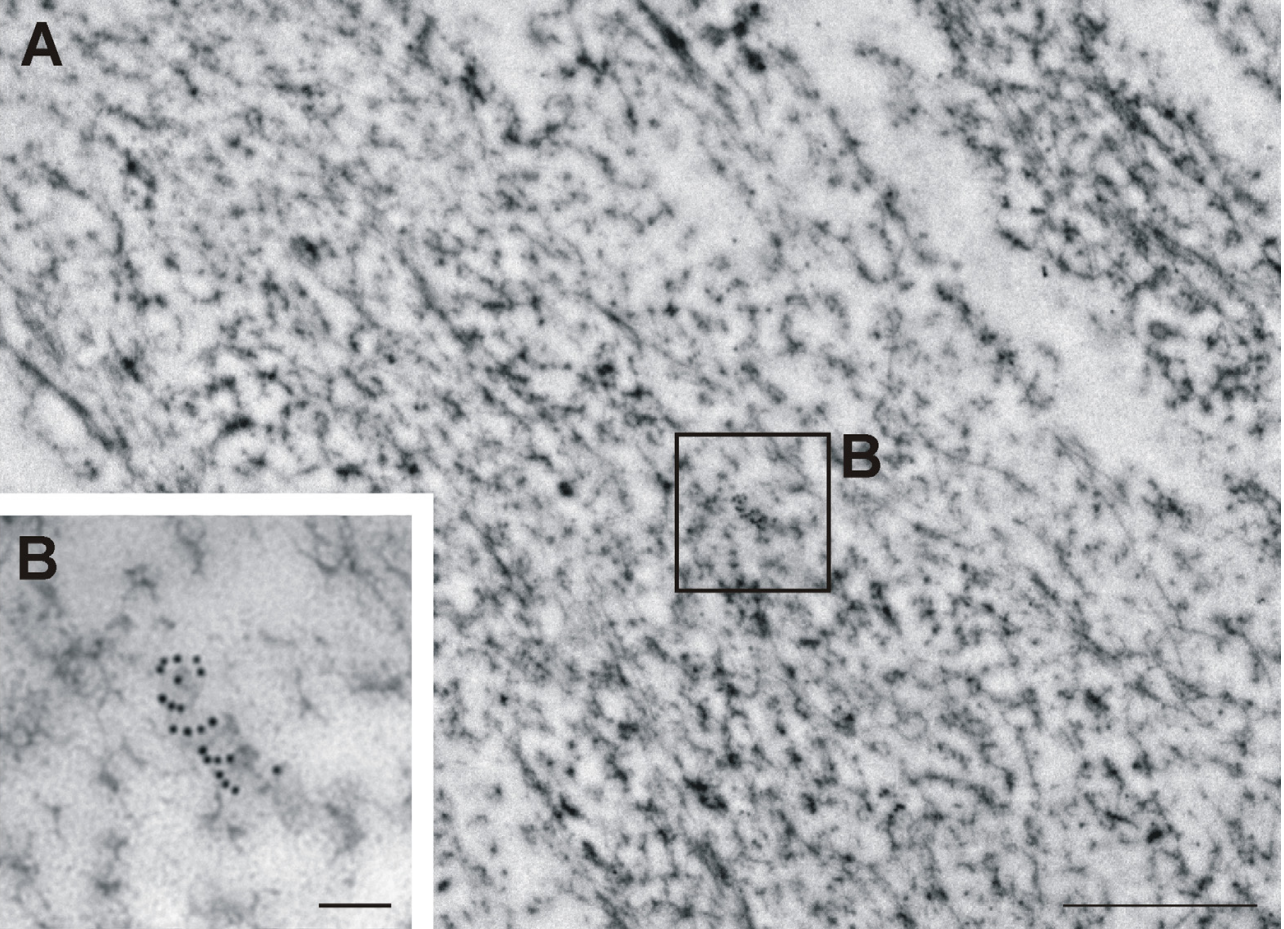
**Labeling of nestin in the glioblastoma cells**. Strong positivity for nestin was detected in the cytoplasm of GM7 cells (A). A special arrangement into short spiral-like fibers in cytoplasm (B) was found repeatedly in different cells. Nestin was detected using immunogold labeling (A–B); bars, 1 μm (A), 0.1 μm (B).

**Figure 7:**
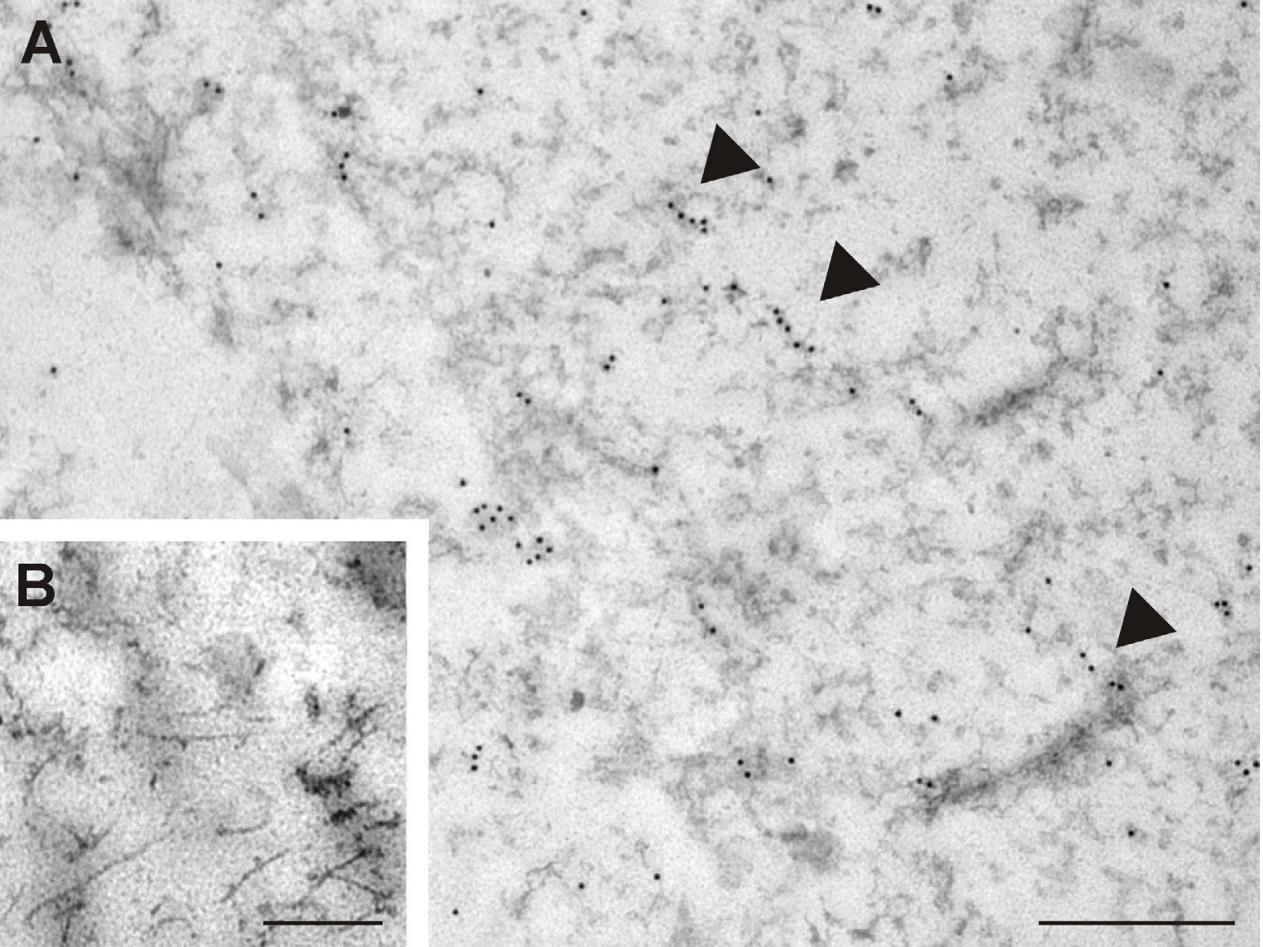
**Ultrastructural distribution of nestin in the cytoplasm of glioblastoma cells**. Labeling of nestin appeared usually as individual signals or small clusters of particles; filamentous structures (arrowheads) were labeled only sporadically in the cytoplasm of GM7 cells (A). Nestin was detected using immunogold labeling; bar, 1 μm (A). Negative control stained with secondary antibody only: cytoplasm; bar, 0.5 μm (B).

**Figure 8:**
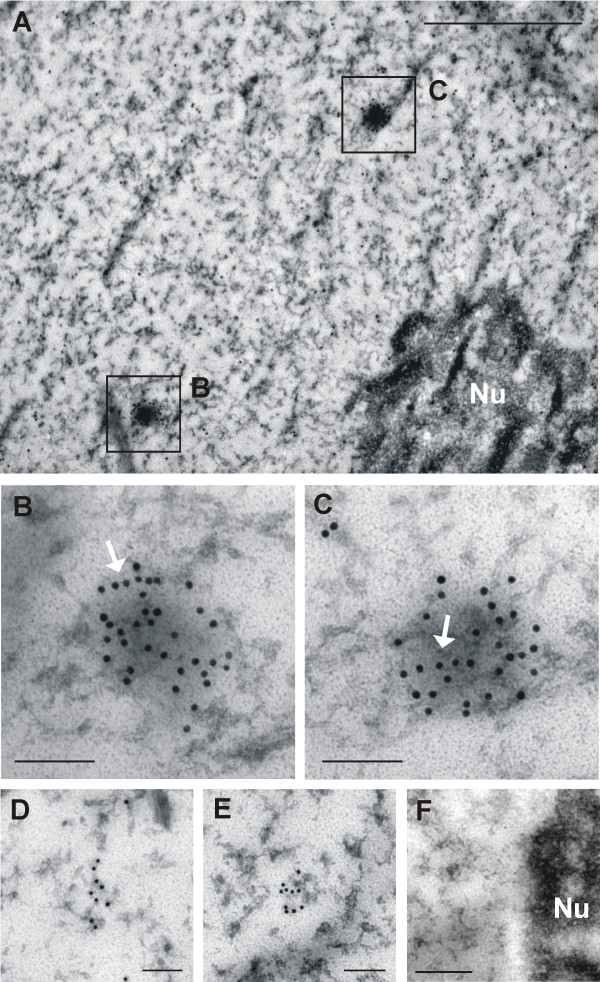
**Detection of nestin in the cell nucleus**. Labeled nestin was distinctly detectable also in the cell nucleus as individual particles and small clusters of signal (A); several larger nestin aggregates (B–C) were also observed. Arrows indicate very short fibers in these aggregates (B–C). A special arrangement into short spiral-like fibers in cell nucleus (D–E) was found repeatedly in different cells. Nestin was detected using immunogold labeling; bars, 1 μm (A), 0.1 μm (B–E). Negative control stained with secondary antibody only: nucleus and nucleolus (Nu); bar, 0.5 μm (F).

Following the immunodetection of nestin in ultrathin sections, a strong nestin positivity was observed throughout the cytoplasm of the tumor cells (Figure 6A). The labeling of nestin usually manifested as individual signals or small clusters of particles; filamentous structures were labeled only sporadically (Figure 7A). In keeping with the findings of epifluorescence and confocal microscopy, nestin was distinctly detectable in the cell nucleus (Figure 8A). In addition to individual particles and small clusters of signal, several large nestin aggregates were also detected in the nucleus (Figure 8A–C) and some very short fibers were also noticeable within these aggregates (Figure 8B,C). A special arrangement of short spiral-like fibers was repeatedly found in both the cytoplasm (Figure 6B) and the cell nucleus (Figure 8D–E).

## Discussion

Our study was focused on the detection and precise morphological characterization of the nestin cytoskeleton in two cell lines derived from glioblastoma multiforme. Nestin was described twenty years ago [1] and it has since been detected in many cell types, both normal and transformed and to this time most of the studies involving nestin have been performed in human tissues using standard histological techniques.

The morphological studies of the nestin cytoskeleton have been carried out primarily in rodent cells. An identical arrangement of cytoskeletal structures in the astrocytes of rat hippocampus suggested a co-polymerization of nestin and vimentin or of nestin and GFAP. Nevertheless, the assumed co-polymerized filaments consisting of nestin and vimentin showed a typical pattern of intermediate filaments, while the co-polymerized filaments from nestin and GFAP had a cytoplasmic microtubules-like appearance [23]. Several authors have described a preferential formation of heteropolymers with vimentin and α-internexin. This is explained as being due to the very short N-terminus of the nestin molecule, which is important for protein assembly and its shortend length leads to less stable nestin homodimers [24,25]. Experiments with targeted mutants confirmed heteropolymers made of nestin and vimentin or nestin and GFAP in mouse cells [26-28]. Co-localization of nestin and vimentin or nestin and desmin filaments, which suggests the formation of heteropolymers, has also been described in chinese hamster ovary cells [29] and in the human fetal myoblast cell line [30].

However, there has been little published information concerning the morphology of intermediate filaments containing re-expressed nestin in human tumor cells. Nestin expression in human brain tumors has been detected by immunohistochemical techniques in a number of studies [4,11-16], and nestin positivity has been detected in the processes of tumor cells in tissue sections [31]. If we assume that nestin can polymerize into filaments with only vimentin, then an identical pattern of intermediate filaments in any given cell is to be expected. This situation was reported in the U-373 MG glioblastoma cell line using the same monoclonal anti-nestin antibody as was used in our study; in these experiments, more details were described in the intermediate filament network after nestin detection [32]. Similar findings, describing the astroglial cells of developing rat neocortex [33], showed more intensive staining of the co-localizing intermediate filaments using monoclonal anti-nestin antibody when compared to anti-vimentin and anti-GFAP antibodies.

In contrast, an incomplete co-localization of nestin with intermediate filament bundles containing other intermediate filament proteins (vimentin, GFAP, neurofilament) has been detected in the cell lines derived from PNETs and malignant gliomas [34]. Likewise, in our cell lines, nestin was detected as a distinct network of intermediate filaments, whereas vimentin and especially GFAP showed a predominantly diffuse positivity in the cytoplasm. Only vimentin was also detected as a filamentous structure, but this network did not completely co-localize with nestin-positive filaments and it was primarily found surrounding cell nucleus. When the same anti-nestin antibody was used, the pattern of intermediate filament positivity was independent of the anti-vimentin antibodies (all mouse monoclonal, but different clones) or the anti-GFAP antibodies (both mouse monoclonal and rabbit polyclonal) that were used for immunostaining. A reduced GFAP-positivity in high-grade astrocytomas has been reported in several studies [35-37] and experiments carried out on the U-373 MG glioblastoma cell line has confirmed a transcription level regulation of both nestin re-expression and GFAP down-regulation [22]. The possible differences in the pattern of nestin-positive and vimentin-positive filaments may be caused by differences in the antibodies that were used for nestin detection. Co-localization of nestin-positive and vimentin-positive filaments was most often reported when rabbit polyclonal antibody was used for nestin detection [26,27,38], whereas, when the mouse monoclonal antibody was used for nestin detection this, usually, produced a more detailed and distinct nestin cytoskeleton pattern [33,37]. In any event, the specifity of the anti-nestin antibody used for detection has to be taken into account [39].

The typical pattern of nestin filament organization, i.e. the asymmetric position of the nestin-positive region in the cytoplasm near the nucleus, which we observed, has also been detected in U-373 and U-251 human glioma cell lines [32,39]. In another study, the nestin cytoskeleton was described as "button-like" clusters in the cytoplasm of anaplastic oligoastrocytoma cells; unfortunately, it is impossible to compare these findings with our results because of the lower magnifications employed compared to the magnifications used in our study [31].

The complete disassembly of the nestin network in mitotic cells, as reported in our experiments, corresponds with the hypothesis that nestin serves as a mediator signal for disassembly of other intermediate filaments during mitosis [29]. Mitotic reorganization of nestin filaments including their partial disassembly has also been detected in the ST15A human neuronal progenitor cell line [40]. Generally speaking, the breakdown of intermediate filaments during mitosis seems to be a common feature of nestin-positive cells [29]. The structural reorganization of the nestin cytoskeleton during the cell cycle is mediated by cdc2 kinase via phosphorylation [40]. Other experiments have confirmed the regulation of nestin organization by phosphorylation on Thr^316 ^and Thr^1495 ^sites that are specific for cdk5 kinase [38]. In general, low levels of nestin phosphorylation are associated with its assembly into filamentous structures [25].

Without question, the most important finding of our study is the discovery of nestin in the cell nucleus, which was unequivocally demonstrated both by fluorescence microscopy and transmission electron microscopy. Even using indirect immunofluorescence, a diffuse signal for nestin in the position of cell nucleus has also been identified in primary cultures of glioblastoma cells [36]. Another study has demonstrated the presence of nestin in the nucleus of neuroblastoma cells by means of cell fractionation and immunoblotting, and other experiments have showed that nestin binds to nuclear DNA in cell lines with N-*myc *amplification [41]. Collectively, all these findings suggest that nestin expression in tumor cells is closely related to their dedifferentiated status and increased malignancy. In addition to the role of the cytoskeletal components in cell growth and motility, which is associated with metastatic potential, some proteins of the intermediate filaments identified in the cell nucleus may affect organization of chromatin or they may serve as specific regulators of gene expression [41,42].

## Conclusion

Using indirect immunofluorescence, we described the re-expression of nestin and the specific morphology of nestin-positive intermediate filaments in glioblastoma cells. Again, the most significant finding is the evidence of nestin in the cell nucleus, which we detected using transmission electron microscopy and immunogold labelling. Although other studies have suggested that changes in the intermediate filament proteins in brain tumors are associated with tumor malignancy and invasiveness, the role of nestin molecules in the nucleus of tumor cells still remains unclear. A thourough study of nestin in the nucleus of tumor cells on the ultrastructural level in combination with a precise molecular cytogenetic characterization of these cell lines will be a main aim of our future research.

## List of abbreviations

BSA, bovine serum albumin; DAPI, 4,6-diamidino-2-phenylindol; DMEM, Dulbecco's modified Eagle's medium; FITC, fluorescein isothiocyanate; GFAP, glial fibrillary acidic protein; MTOC, microtubule-organizing center; PBS, phosphate-buffered saline; PNETs, primitive neuroectodermal tumors; TRITC, tetramethylrhodamine isothiocyanate

## Competing interests

The author(s) declare that they have no competing interests.

## Authors' contributions

RV conceived the study, carried out the cytoskeleton analysis and drafted the manuscript. PK carried out the cytogenetic analysis and participated in manuscript preparation. PC performed the operations of patients and managed the COST project. HS participated in the immunofluorescence and electron microscopy studies. JN and TL were involved in the electron microscopy study; the former besides took part in the manuscript preparation. JR coordinated this study and managed the VZ project.

## Pre-publication history

The pre-publication history for this paper can be accessed here:


